# Acute thyroid swelling after fine needle aspiration—a case report of a rare complication and a systematic review

**DOI:** 10.1186/s12893-021-01160-z

**Published:** 2021-03-31

**Authors:** Tiantong Zhu, Ye Yang, Hao Ju, Ying Huang

**Affiliations:** Department of Ultrasound, Shengjing Hospital of China Medical University, 36 Sanhao Street, Heping District, Liaoning Shenyang city, China

**Keywords:** Fine needle aspiration, Complication, Thyroid swelling, Anaphylactic reaction

## Abstract

**Background:**

Thyroid fine needle aspiration (FNA) is the procedure of choice in the management of thyroid nodules. Acute thyroid swelling after FNA is a rare complication and is reported in a finite number of literatures. To the best of our knowledge, only seven reported cases exist in literatures. This study describes an addition case with an acute thyroid swelling after FNA, as well as puts forward a new hypothesis of this phenomenon.

**Case presentation:**

The case is presented of a 30-year-old female with an acute thyroid swelling after FNA, with funicular hypoechoic lesions in thyroid gland. The size of thyroid was 1.5-fold enlarged in the unilateral thyroid gland. No complains of pain or other discomforts with her and no signs of hemorrhage were found along the passage of the fine needle. The episode was recovered spontaneously.

**Conclusions:**

An acute thyroid swelling is a rare complication of FNA. A hypothesis of anaphylactic reaction was suggested in our study. Physicians should pay more attention of this phenomenon and more information is needed to support our hypothesis.

## Background

Thyroid fine needle aspiration (FNA) is the procedure of choice in the management of thyroid nodules. Although invasive, FNA is simple, reliable, and except for slight pain or discomfort at the site of aspiration, complications seem to be rare [1, 2]. Acute thyroid swelling is a rare complication and is reported in a finite number of literatures. Several explanations of the main reason have been suggested but cannot be clarified [3]. Here, the case is described of an acute thyroid swelling after FNA, accompanied with funicular hypoechoic lesions in thyroid gland. A new hypothesis is proposed that it is the anaphylactic reaction resulting in this phenomenon.

## Case presentation

**Fig. 1:**
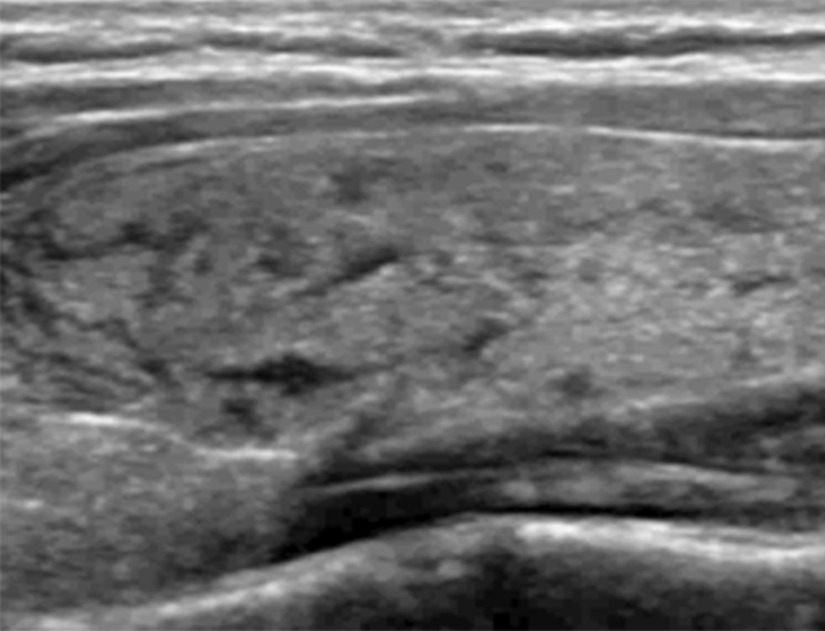
Ultrasonography showed the left thyroid lobe was swelling with multiple funicular hypoechoic lesions

A thirty-year-old woman referred to the hospital because of nodules in her bilateral thyroid lobes. Ultrasonography showed a heterogeneously echoic (iso/hypoechoic) nodule (13 × 8 × 9 mm) with multiple microcalcifications in left lobe and a hypoechoic nodule (5 × 5 × 5 mm) in right lobe. There was neither known allergies nor use of specific medication. Before FNA, a conventional ultrasound and contrast-enhanced ultrasound was performed. 2 mL of SonoVue (Bracco, Italy) suspension (mixed by 5 mL saline and 39 mg SonoVue powder) was administered manually. Her skin of neck was sensitive to turn red after being contacted by the probe. Subsequently, 0.5 ml Lidocaine solution was applied for surface anesthesia. FNA of the nodule in the superior pole of right lobe was performed with a 23-guage needle. As soon as pulling out the needle, an obvious local swelling with multiple funicular hypoechoic lesions was observed in the inferior pole of left thyroid lobe. It lasted for only several seconds. Ten minutes later, FNA of the nodule in left thyroid lobe was performed and similar episode came out immediately after the procedure. The left thyroid lobe was swelling with multiple funicular hypoechoic lesions and enlarged to 1.5-fold increase in contrast with the size before. (Fig. 1) The targeted nodules had nothing different with before. The hypoechoic lesions faded away in thirty minutes without compression. The patient had no complains of pain and dyspnea. No signs of hemorrhage were noted along the passage of the needle. Cytologic diagnosis showed a benign one in the left thyroid lobe and atypical undetermined significance (AUS) in the right thyroid lobe.

## Discussion and conclusions

Acute swelling of thyroid is rare and has been reported only in a small number of literatures. It was first reported by Haas. in 1982 [4], with a nearly 2.5-fold increase of the thyroid volume following FNA, being described as a frightening episode. Dal Fabbro et al. (in 1987) [5]and Van den Bruel et al. (in 2008) [6] reported acute transient thyroid swelling with with slightly or acute pain. In 2011, Norrenberg S et al. [3]reported two cases of acute swelling with acute pain over the 20-years process of FNA procedure. The episode lasted for 1 or 3 h. In 2016, Uchida et al. [7] reported an acute transient thyroid swelling and recovered after hydrocortisone treatment. Bouwman [8]reported the episode occurred after several unsuccessful subclavian vein catheterization attempts in 2009. And the author suggested that it was because cephalad needle direction contributed an advertent needle into the thyroid gland. With our case, all of the eight cases showed consistent in ultrasound appearance and 1.5- to 3-fold enlarged of thyroid gland. The episode happened immediately or shortly after FNA. Most cases accompanied with slightly to moderately pain, one of them had choking and one of them was associated with airway obstruction. Different with other cases, the patient in our case did not complain any discomfort. Some cases were treated by analgesic-antipyretics or hydrocortisone, some of them regressed spontaneously. The episode lasted from a few minutes to hours. All of the patients do not show common medical history, clinical manifestations, laboratory examination and pathological results. No uniform drugs were applied. Needles of different sizes were utilized (from 21- to 24-gauge and one subclavian catheter). (Table 1)

**Table 1:** Features of thyroid swelling after FNA were mentioned in literatures

Year	Size of Enlarged	Time of occurrence	Management	Time of duration	Other Symptoms	Pathological result	Surface medication	Needle
1982 [4]	2.5-fold	2 to 3 min after FNA	Cold packs	1 h	Moderately tender	Class I negative	Lidocaine	22-guage
1987 [5]	3-fold	Immediately after the withdrawal of needle	Cold packs	A few hours	Slightly pain	Follicular carcinoma	No	22-guage
2008 [6]	1.5- to 3-fold	After the second pass	Without	1 h	Acute pain	Medullary carcinoma	Not mentioned	24-guage
2009 [8]	Not mentioned	Shortly after a subclavian vein catheterization attempt	Without	Within 4 h	Airway obstruction	Absent	Not mentioned	Not mentioned
2011 [3]	2-fold	After the second aspiration	Diclofenac	3 h	Acute pain	Absent	Chlorhexidine	21-guage
2011 [3]	1.5-fold	5 min after the first aspiration	paracetamol	At least 1 h	Acute pain	Benign cystic colloid nodule	Chlorhexidine	21-guage
2016 [7]	Not mentioned	Shortly after FNA	Hydrocortisone	At least 40 min	Choking	Benign	No	22-guage

The underlying mechanism of acute swelling is not clear yet. Haas did not propose any hypothesis [4]. Dal Fabbro et al. analyzed the cause of it but failed to explain it [5]. They suspected blood causing the swelling, but the negative findings questioned the suspicion. They did not believe an inadvertently injection of air as no signs of emphysema could be detected. In our case, an intrathyroidal bleeding is first suspected but it can be excluded by the following reasons. (a) In our case, the puncture point was in superior pole of right thyroid lobe, but the patchy hypoechoic lesions came out in inferior pole first. (b) No actively bleeding site was observed along the way of the needle passage and no evidence of hematoma formation. (c) No feelings of swelling pain or other discomfort were complained. (d) Without any compression it regressed spontaneously. All of these clues could not support the hypothesis of bleeding. Van den Bruel et al. also excluded this hypothesis on retrospect due to the hyperacute swelling, which had a reversible nature within 1 h [6].

Van den Bruel et al. [6]previously proposed the theory of vasodilation and capillary leaking. They hypothesized Calcitonin Gene Related Peptide (CGRP) could be responsible for the observed phenomenon. Immunostaining for CGRP was positive in their cases but CGRP almost universally expressed by medullary thyroid cancer. Sarah Norrenberg et al. also suggested the proposal of CGRP could not explain the phenomenon in other cases [3].

From our perspective, this rare episode was hypothesized as a kind of anaphylactic reaction even though the hypothesis was ruled out by previous literatures. Sarah Norrenberg et al. [3] proposed that allergy could not explain this phenomenon because no signs of allergy were observed and no common solution was in contact with the skin or the gland tissue. Dal Fabbro [5] suggested that an allergic etiology seems unlikely, owing to the extremely rapid onset of swelling and to the fact that they did not inject any drug. When the episode occurred in our case, an anaphylactic reaction was suspected by the cause of the contrast-enhanced agents. The polymer of the encapsulated shell of microbubbles could be a foreign antigen to human body. But the hypothesis could not be applied in other cases. The main reason of this phenomenon may be not foreign materials, such as drugs, but the innate molecules. FNA can trigger biochemical alterations in serum, since it may destroy thyroid follicles, resulting in thyroglobulin release into the circulation [9]. May be the thyroglobulin or other molecules provoke an anaphylactic reaction of thyroid itself. On account of the sensitive skin of the patient, we highly suspected the possibilities of the hypersensitivity reaction even without allergic history. An allergic reaction is rapid in onset and could result in dilation of vessels and an increase of vascular permeability, which is supported by the hypothesis of previous literature [2]. The ultrasound appearance, a transient homogeneous echoic lesion in the extranodular thyroid parenchyma, could be a proof of this hypothesis. Many of those who have experienced anaphylaxis have survived the episode without any treatment [10]. The feature of self-limiting in reported episode keeps consistent with the characteristic of anaphylaxis reaction.

In conclusion, acute thyroid swelling after FNA of the thyroid gland is a rare complication. Ultrasonography showed thyroid lobes enlarged from 1.5 to 3-fold with funicular hypoechoic lesions accompanied with pain or not. The self-limiting course lasted for 30 min to 4 h, which is not life-threatened. No exact pathology or etiology has been convinced. We proposed a hypothesis of anaphylactic reaction but can’t be verified by laboratory data. We hope ultrasound physicians could pay attention to this rare complication and manage the frightened situation appropriately. More information is needed to support our hypothesis.

## Data Availability

Not applicable.

## Abbreviations

FNA: Fine needle aspiration
CGRP: Calcitonin Gene Related Peptide
AUS: Atypical undetermined significance
